# Supplementary material for: Effectiveness of Deep Water Running on Improving Cardiorespiratory Fitness, Physical Function and Quality of Life: A Systematic Review
Source: Int J Environ Res Public Health. 2022 Aug 1;19(15):9434. doi: 10.3390/ijerph19159434 (PMC9367787; doi:10.3390/ijerph19159434)
Supplement: Supplementary file 1 [file ijerph-19-09434-s001.zip › ijerph-1807119-supplementary.pdf]

Table S1. Search Strategy

Databases	Main search terms
SPORTDiscus,<br>MEDLINE,<br>CINAHL,<br>AMED,<br>Embase, and<br>The Cochrane Library	‘deep water run*’ or ‘deep water jog*’ or ‘aqua jog*’ or ‘aqua run*’ or ‘running under water’ or ‘running in water’ or ‘jogging under water’ or ‘jogging in water’.

Table S2. Eligibility Criteria

**Inclusion Criteria**
Study Characteristics
– Adults (aged above 18)
– Longitudinal studies (either randomised or non-randomised trials)
– Studies must include comparison group(s)
– Measurable outcomes in either cardiorespiratory fitness gait performance or quality of life
Report Characteristics
– Written in English
– In full text in peer-reviewed journals
**Exclusion Criteria**
Study Characteristics
– Non-experimental studies
– Cross-sectional studies
– Not DWR exercise
– Combined exercise or treatments in intervention group
– Measurable outcomes on biomechanics of gait, strength, balance, or anthropometric parameters
Report Characteristics
– Any reviews, unpublished articles

Table S3. Selected components from Downs and Black's Checklist for measuring study quality

Subscale	Items
Reporting	1. Is the hypothesis/aim/objective of the study clearly described?
	2. Are the main outcomes to be measured clearly described in the intro or methods section?
	3. Are the characteristics of the patients included in the study clearly described?
	4. Are the interventions of interest clearly described?
	5. Are the distributions of principal confounders in each group of subjects to be compared clearly described?
	6. Are the main findings of the study clearly described?
	7. Does the study provide estimates of the random variability in the data for the main outcomes?
	10. Have actual probability values been reported (e.g. 0.035 rather than <0.05) for the main outcomes except where the probability value is less than 0.001?
External validity	11. Do the subjects asked to participate represent the population?
	12. Do the subjects in the study represent the populations?
Internal validity -Bias	15. Was an attempt made to blind those measuring the main outcomes of the intervention?
	18. Were the statistical tests used to assess the main outcomes appropriate?
	20. Were the main outcome measures used accurate (valid and reliable)?
Internal validity -Confounding	23. Were study subjects randomised to intervention groups?
	24. Was the randomised intervention assignment concealed from both patients and health care staff until recruitment was complete and irrevocable?
	26. Were losses of patients to follow-up taken into account?
Power	27. Did the study have a power calculation?

Table S4. Benefits of DWR among three target populations

Population	Purpose	Benefits of DWR	Evidences
Aerobically trained athletes			
- Injured runners	Rehabilitation	- Reduces deconditioning<br>- Accelerates rehabilitation while maintaining high aerobic conditioning<br>- Maintains aerobic capacity while reducing mechanical load on lower limbs	***Findings from included studies***<br>- Runners maintained similar VO_2max and 2-mile run time with a 6-week DWR training for rehabilitation[1].<br>***Findings from previous publications***<br>- Simulates running in water without incurring possible harmful effects due to weightbearing[2].<br>- Reduces mechanical load on lower limbs[2].
- Uninjured athletes	Supplementary training<br>Alternative training<br>Strength and conditioning training	- Minimizes likelihood of getting injured<br>- As light recovering workout following high intensity trainings<br>- As recovery from delayed-onset muscle soreness brought by land-based training<br>- Prevents detraining<br><br>**Application:**<br>- Cross training	***Findings from included studies***<br>- Non-injured athletes showed no significant difference in VO_2max following 6-week DWR in comparison with treadmill running and land-based running[2].<br>- DWR workout served as an effective alternative training[1-3].<br>***Findings from previous publications***<br>- DWR serves as light recovering workout following high intensity trainings[1-3].<br>- DWR as cross training for runners[3, 4].
Individuals with health conditions			
- Obesity	Aerobic training	- Improves body composition, blood pressure, and fitness status<br>- Weight reduction	***Findings from included studies***<br>- Increases energy expenditure for weight reduction[5].<br>- Reduces mechanical loads on joints[5].<br>***Findings from previous publications***<br>- ACSM suggested aquatic aerobic exercise like DWR as favourable choice of aerobic training [5, 6].<br>- Enhances physical function with lower injury risk[5, 6].<br>- Increases adherence to exercise training for inactive individuals[5, 6].
Other health conditions:<br>- Chronic low back pain<br>- Fibromyalgia	Non-pharmacologic pain management	- Activates different trunk muscles<br>- Stabilization in lumbopelvic complex<br>- Reduces pain<br>- Reduces kinesiophobia	***Findings from included studies***<br>- DWR significantly reduce VAS in subjects with fibromyalgia and low back pain with moderate to high effect size[7-9].<br>- Properties in warm aquatic environment promotes relaxation, vasodilation and analgesic effects for fibromyalgia patients[9].

Population	Purpose	Benefits of DWR	Evidences
	Safe therapeutic exercise	- Affects quality of life<br>- Provides safer environment<br>- Gains in cardiorespiratory fitness<br><br>***Application:***<br>- Suitable for improving dynamic trunk stability and pain reduction	***Findings from previous publications***<br>- Buoyancy lessens axial load on spine, while unstable aquatic environment acts as a challenging component to maintain trunk stability[10].<br>- Feet non-contacting floor during DWR fosters coordination and stabilization in lumbopelvic complex[8].<br>- Exercise helps to release cortisol and adrenaline into bloodstream and therefore increase pain threshold[8].<br>- Hydrostatic pressure exerted on skin triggers mechanoreceptors that helps blocking nociceptors[10].<br>- Immersion in warm water speeds up body metabolism, for faster removal of metabolic waste and activates the nociceptors[11].
Untrained population			
- Community dwelling elderly	Aerobic training<br>Strength training	- Increases cardiac output and stroke volume by aquatic immersion<br>- Allows interval aerobic exercise at high loads with low risk of injury	***Findings from included studies***<br>- Elderly subjects showed increased power in lower extremity and better physical function[12].<br>- improvements in submaximal and maximal aerobic power among elderly women after 8-week high intensity interval DWR with vest[13].<br>***Findings from previous publications***<br>- Gains in aerobic fitness for elderly[14].<br>- Aquatic immersion facilitates central shift of blood volume due to hydrostatic pressure[15].<br>- Flotation vest keeps the body in upright position and avoid contact with the floor[16].
- Sedentary healthy population	Aerobic training	- Gains in cardiorespiratory fitness<br><br>***Application:***<br>- Suitable for low impact and less thermally stressful training	***Findings from included studies***<br>- Improvement in VO2max in DWR almost twice as much as treadmill running[3].<br>- DWR as non-weightbearing activity adopted different muscle recruitment pattern [3].<br>- exhausting less work on large muscle groups in lower extremity[3].<br>- more work for upper extremity during arm and shower movements[3].

ACSM: American College Sports Medicine

References

1. Eyestone, E.D., et al., *Effect of water running and cycling on maximum oxygen consumption and 2-mile run performance.* The American journal of sports medicine, 1993. **21**(1): p. 41-44.
2. Wilber, R.L., et al., *Influence of water run training on the maintenance of aerobic performance.* Medicine and Science in Sports and Exercise, 1996. **28**(8): p. 1056-1062.
3. Michaud, T.J., et al., *Aquarunning and gains in cardiorespiratory fitness.* The Journal of Strength & Conditioning Research, 1995. **9**(2): p. 78-84.
4. Davidson, K. and L. McNaughton, *Deep water running training and road running training improve VO2max in untrained women.* Journal of Strength & Conditioning Research, 2000. **14**(2): p. 191-195.
5. Colato, A., et al., *Impact of aerobic water running training on peripheral immune-endocrine markers of overweight-obese women.* Science and Sports, 2017. **32**(1): p. 46-53.
6. American College of Sports Medicine, *ACSM's guidelines for exercise testing and prescription*. 9th ed. Exercise prescription for healthy populations with special considerations and environmental considerations. 2014, Philadelphia: Lippincott Williams & Wilkins.
7. Kanitz, A.C., et al., *Aquatic and land aerobic training for patients with chronic low back pain: a randomized study.* Human Movement, 2019. **20**(1): p. 1-8.
8. Cuesta-Vargas, A., et al., *Deep water running and general practice in primary care for non-specific low back pain versus general practice alone: randomized controlled trial.* Clinical Rheumatology, 2012. **31**(7): p. 1073-1078.
9. Assis, M.R., et al., *A randomized controlled trial of deep water running: clinical effectiveness of aquatic exercise to treat fibromyalgia.* Arthritis Care & Research, 2006. **55**(1): p. 57-65.
10. Kanitz, A.C., et al., *Effects of two deep water training programs on cardiorespiratory and muscular strength responses in older adults.* Exp Gerontol, 2015. **64**: p. 55-61.
11. Hall, J., et al., *Does Aquatic Exercise Relieve Pain in Adults With Neurologic or Musculoskeletal Disease? A Systematic Review and Meta-Analysis of Randomized Controlled Trials.* Archives of Physical Medicine and Rehabilitation, 2008. **89**(5): p. 873-883.
12. Alberti, D., L. Lazarotto, and P.C.B. Bento, *Effects of a deep-water running program on muscle function and functionality in elderly women community dwelling.* Motriz: Revista de Educação Física, 2017. **23**(4): p. 1-8.
13. Broman, G., et al., *High intensity deep water training can improve aerobic power in elderly women.* European Journal of Applied Physiology, 2006. **98**(2): p. 117-123.
14. Jorgic, B., et al., *Effects of deep water running in older adults. a systematic review.* HealthMED, 2012. **6**(9): p. 3219-3227.
15. Reilly, T., C.N. Dowzer, and N.T. Cable, *The physiology of deep-water running.* Journal Of Sports Sciences, 2003. **21**(12): p. 959-972.
16. Kanitz, A.C., et al., *Effects of two deep water training programs on cardiorespiratory and muscular strength responses in older adults.* Experimental Gerontology, 2015. **64**: p. 55-61.
